# Lessons from COVID-19 patient visitation restrictions: six considerations to help develop ethical patient visitor policies

**DOI:** 10.1007/s40592-025-00230-9

**Published:** 2025-02-08

**Authors:** Tracy Beth Høeg, Benjamin Knudsen, Vinay Prasad

**Affiliations:** 1 Sloan School of Management, Massachusetts Institute of Technology, Office E62-562. 100 Main St., Cambridge, MA 02142 USA; 2 Department of Clinical Research, University of Southern Denmark, Odense, Denmark; 3 Department of Emergency Medicine, University of California-San Francisco, San Francisco, USA; 4 George Washington University School of Medicine and Health Sciences, Washington, DC USA; 5 Department of Epidemiology and Biostatistics, University of California-San Francisco, San Francisco, USA

**Keywords:** Patient visitor restrictions, COVID-19, Infectious diseases, Health policy, Medical ethics

## Abstract

Patient visitor restrictions were implemented in unprecedented ways during the COVID-19 pandemic and included bans on any visitors to dying patients and bans separating mothers from infants. These were implemented without high quality evidence they would be beneficial and the harms to patients, families and medical personnel were often immediately clear. Evidence has also accumulated finding strict visitor restrictions were accompanied by long-term individual and societal consequences. We highlight numerous examples of restrictions that were enacted during the COVID-19 pandemic, including some that continue to be in place today. We outline six specific concerns about the nature and effects of the visitor restrictions seen during the COVID-19 pandemic. These considerations may help provide both an ethical and science-based framework, through which healthcare workers, families and government entities can work towards safeguarding patient and family rights and well-being.

## Introduction

### Historical background and evidence review

Hospital visitor restrictions have been implemented in varying ways and for a variety of reasons since at least the 1700s (Ismail and Mulley 2007). The evidence for their general effectiveness against transmission of upper respiratory viruses is mixed and overall weak (Smith et al. 2009; Hugelius et al. 2021). Very few studies have analyzed the effectiveness of visitor restrictions alone on the prevention of respiratory infections.

One quasi-experimental study at a children’s hospital performed prior to the COVID-19 pandemic found that capping visitors to three in the intensive care unit and four on general wards, limiting young children (< 12 years) and anyone with upper respiratory infection symptoms was associated with a significantly decreased ratio of hospital-acquired illness to community-acquired illness during the periods of restriction (Forkpa et al. 2020). However, a more recent study from a large hospital system with 2280 beds in Singapore, evaluated the relaxation of COVID-19 visitor restrictions alone without accompanying changes in other policies (Wee et al. 2021). This latter study did not find either of the two periods where restrictions were relaxed—from no visitors to one or from one to two—to be associated with a rise in hospital-associated respiratory viral illnesses or hospital-associated SARS-CoV-2 transmission.

A retrospective observational study performed during 2014–2016 at a Cincinnati Children’s Hospital found that standardizing visitor restriction policy to a fixed list of six nonparent, nonlegal guardian persons was associated with a 37% reduction (60 vs. 37 infections) in the incidence of hospital acquired respiratory viral infections during the winter viral respiratory period (Washam et al. 2018). On the contrary, in this same study and time period, the intensive care unit (ICU) visitation policy was relaxed, from a four to six visitor cap, and no accompanying significant difference was observed (Washam et al. 2018). However, the findings may have been confounded by a milder viral respiratory season during the intervention period. This study highlights the challenges of drawing causal conclusions from observational studies about the effectiveness of visitor restrictions. Well-designed randomized trials would minimize confounding differences between groups and be more appropriate for establishing the effectiveness or lack thereof of visitor restriction policies.

We identified two randomized controlled trials (RCTs) exploring this topic (Rosa et al. 2019; Fumagalli et al. 2006). The first failed to find a reduction in ICU-acquired infections by limiting visitation hours from 12 to 1.5 h per day. Notably, this same RCT also found family anxiety and depression scores to be significantly increased with more restrictive visitation policies (Rosa et al. 2019). The second trial found less restrictive ICU visitation to have a significantly higher microbial contamination rates but no accompanying increase in infectious complications (Fumagalli et al. 2006). Patients randomized to only one visitor for a total of 1 h a day had a twofold higher cardiocirculatory complication rate, persistently higher anxiety scores and a non-significant 2.9-fold higher all-cause mortality rate (Fumagalli et al. 2006). A small systematic review and meta-analysis performed in 2017 found less restrictive ICU visitation policies were *not* associated with higher all-cause mortality, ICU-acquired infection or longer ICU stay (Nassar Junior et al. 2018). Though it may seem logical that restricting hospital visitors would decrease in-hospital respiratory infection risk, it is important to note this has not been consistently borne out in the literature and existing randomized studies point to overall worsening of patient outcomes with stricter visitation policies.

## COVID-19-specific visitor restrictions

COVID-19 patient visitation restrictions occurred in the United States to unprecedented degrees (Sudai 2021; Romano-Keeler et al. 2021) and, in some places, continue to this day, albeit in less strict forms. The Centers for Disease Control and Prevention (CDC) made a non-specific recommendation in March of 2020 to limit visitors to inpatient facilities to those “essential for the patient's physical or emotional well-being and care.” (Centers for Disease Control and Prevention (CDC) 2020).

However, data on hospital policy changes suggest (Darcy Mahoney et al. 2020) the majority of hospitals had already increased restrictions by the time the CDC released its guidelines. Across the United States, hospitals and long-term care facilities enacted policies, which resulted in patients dying alone (Sudai 2021), mothers being separated from infants (Romano-Keeler et al. 2021; Darcy Mahoney et al. 2020) and children being hospitalized without any visitation allowed from parents or guardians (Kitano et al. 2020).

Although some documentation of hospital visitation policies during the COVID-19 pandemic is still available online today, numerous records are no longer obtainable or only available through web archive searches when the date and uniform resource locator (URL) of the policy are known. Thus, there is no comprehensive database or systematic method available to analyze nationwide policies. This makes it challenging to characterize the nature or duration of COVID-19 visitation restrictions across the United States. We performed PubMed and Google searches and supplemented these searches with information from Web Archives when older but relevant online documents were no longer available. Because we were unable to describe the entire landscape of restrictions across the United States, in Table 1, we feature select examples of policies enacted in various states, which were noteworthy because of how strict they were and/or that continue to this day.

**Table 1:** Select examples of healthcare visitor limitations during the COVID-19 pandemic

Effective through at least	Hospital, location	Policy
March 2020	Hospitals in multiple states: NY, WA, CA, WI	Families reporting to the news media their loved ones died alone and no visitors were permitted^a^
March 2020	UCSF, San Francisco, CA	No visitors regardless of COVID-19 status of patient or family, including for young people with life-threatening conditions^b^
April 2020	7 Neonatal ICUs in the United States	7 NICUs (hospital names not disclosed) did not allow any visitation from parents and 2/7 did not allow parents at the bedside even if infant was felt to be in critical/terminal condition^c^
June 2020	Veterans Affairs (VA), multiple states	Policies varied but 27% of family members reported that they were not allowed to enter the VA to see the patient at the end of life in spite of official VA policy allowing “compassionate care” visits for dying patients^d^
June 2020	CA, IA, TN, IL, NY	No visitors, including parents and guardians, of hospitalized children^e^
June 2020	UPMC, Pittsburgh, PA	“UPMC is temporarily restricting visitation at all UPMC Senior Communities long-term care facilities, including skilled nursing, personal care, and assisted and independent living settings, and hospital transitional care units (TCUs). Visitors will be permitted only in special situations, approved by the facility administrator or designated manager. Special situations may include end-of-life visitation and when a visitor is essential for the resident’s emotional well-being and care^f^”
July 2020	University of Rochester, Rochester, NY	“Visitation for Patients Admitted to the Hospital: No COVID-19-positive or quarantined patients may have visitors unless they are deemed an exception at the discretion of the provider^i^”
July 2020	Intermountain Healthcare	For end-of-life or deceased patients who are COVID-positive or PUI The number of visitors will be limited to 2. Those visitors will be designated for all encounters and cannot switch with others “Although death is a time to support each other with compassion, due to viral transmission, visitors should not hug or physically comfort patients or other family members at this time.” Visitation period is limited to 1 h or less to reduce risk of exposure^g^”
August 2020	University of Illinois, Chicago	Infants born to COVID-19 positive moms separated from their mother. Skin-to-skin contact with mother avoided. No visitors/parents allowed. Discharge arranged with COVID-19 negative caretakers^h^
December 2021	MedStar Health – Georgetown, DC	“General guidance for hospital and ambulatory locations No Visitors Permitted at Hospitals for patients > 18 years. Visitation exceptions for Persons Under Investigation (PUI) or COVID-19 positive patients must be approved by hospital Chief Medical Officer or Chief Nursing Officer (or their designee)^l^”
April 2023	States of California (State guidelines)	Parents/guardians not allowed to visit hospitalized children without primary vaccination series or proof of negative COVID-19 test^j^
April 2023	UPMC, Pittsburg, PA	Children down to age 2 are not allowed to visit patients in the hospital or accompany parents in clinic unless they are able to wear a mask^k^

^a^Sudai M. Not Dying Alone: the Need to Democratize Hospital Visitation Policies During Covid-19. *Med Law Rev*. 2021;29(4):613–638. 10.1093/medlaw/fwab033

^*b*^*'a heart-wrenching thing': Hospital bans on visits devastate families*. The New York Times. Retrieved April 24, 2023, from https://www.nytimes.com/2020/03/29/health/coronavirus-hospital-visit-ban.html

^c^Darcy Mahoney A, White RD, Velasquez A, Barrett TS, Clark RH, Ahmad KA. Impact of restrictions on parental presence in neonatal intensive care units related to coronavirus disease 2019. *J Perinatol*. 2020;40(Suppl 1):36–46. 10.1038/s41372-020-0753-7

^d^Feder S, Smith D, Griffin H, et al. "Why Couldn't I Go in To See Him?" Bereaved Families' Perceptions of End-of-Life Communication During COVID-19. *J Am Geriatr Soc*. 2021;69(3):587–592.

^e^Vance AJ, Duy J, Laventhal N, Iwashyna TJ, Costa DK. Visitor Guidelines in US Children's Hospitals During COVID-19. *Hosp Pediatr*. 2021;11(6):e83-e89. 10.1542/hpeds.2020-005772

^f^*Covid-19 visitor restrictions*. UPMC. (n.d.). Retrieved April 23, 2023, from https://www.upmc.com/coronavirus/visitor-restrictions

^g^*End-of-life visitation guidelines—intermountain healthcare*. (n.d.). Retrieved April 24, 2023, from https://intermountainhealthcare.org/-/media/files/covid-caregiver/08-14-2020/policy_visitors-dying-deceased-071520.pdf?la=en

^h^Romano-Keeler J, Fiszbein D, Zhang J, et al. Center-Based Experiences Implementing Strategies to Reduce Risk of Horizontal Transmission of SARS-Cov-2: Potential for Compromise of Neonatal Microbiome Assemblage. Preprint. *medRxiv*

^i^*Visitor policies and guidance during covid-19*. Wayback Machine. (n.d.). Retrieved April 24, 2023, from https://web.archive.org/web/20200926213653/https://www.urmc.rochester.edu/MediaLibraries/URMCMedia/hh/pdf/covid-19-2020-limited-visitation7-20.pdf. Accessed 4/23/2023

^j^*Covid-19 information and updates*. COVID-19 Information and Updates. (n.d.). Retrieved April 23, 2023, from https://www.shrinerschildrens.org/en/locations/northern-california/patients-and-families/visiting-us/coronavirus

^k^*Covid-19 visitor restrictions: UPMC*. UPMC | Life Changing Medicine. (n.d.). Retrieved April 24, 2023, from https://web.archive.org/web/20200715165343/https://www.upmc.com/coronavirus/visitor-restrictions

Noteworthy examples include seven ICUs that prohibited parents from visiting children in the NICU, with 2/7 prohibiting parents from being with sick and dying newborns (Darcy Mahoney et al. 2020). The University of Illinois separated COVID-19 positive mothers from infants, prohibiting skin to skin contact (Romano-Keeler et al. 2021). Intermountain Health specifically advised visitors, “Although death is a time to support each other with compassion, due to viral transmission, visitors should not hug or physically comfort patients or other family members at this time.”

While hospital systems websites often no longer contain former policies, news outlets continue to have older articles available which describe the coercive nature of these policies in critical or end of life moments. One report quoted a daughter of a dying patient saying, “as her father, Gary Young, died of coronavirus in an isolation ward on Tuesday, Stacey stood in the ICU hallway, behind two sets of glass doors, and watched as the medical team… finally turned off the heart monitor. ‘It broke my heart into a million pieces. I didn’t want him to feel alone…But there was nothing I could do.’” (Julia Prodis Sulek 2020) As one physician said, “So often a patient will be on their deathbed, dying alone, and it’s been incredibly painful to see the suffering of family members who I call from the ICU, hearing the tears, crying with them on the phone.” (Anon 2020).

## Six considerations to inform future patient visitor restriction policies

Many providers and patients accepted the unprecedented COVID-19 restrictions as necessary for the “greater good” (Sudai 2021) or as simply “unavoidable reality.” (Ramos 2020). However, these concessions ignore the ethical issues raised by and the potential societal and health consequences of these experimental policies.

In the remainder of this paper, we outline six considerations that we hope can help inform future policy making. These include (1) The benefits of stricter policies have not been shown to outweigh the harms, (2) Harming one patient because it may benefit others is not consistent with basic medical ethics, (3) Policies enacted by healthcare administrators may be too narrowly focused, (4) Fundamental patient rights should be legally protected, (5) The loss of the ability to consent is a form of coercion and (6) Moral injury and dehumanization in healthcare may result from unscientific or visibly harmful policies.

1. The benefits of stricter policies have not been shown to outweigh the harms

Existing high-quality evidence has not demonstrated the benefits of patient-visitor restrictions outweigh the risks (Rosa et al. 2019; Fumagalli et al. 2006; Nassar Junior et al. 2018). Randomized studies specific to COVID-19 were not done but could have been performed during the pandemic (Iness et al. 2022). The lack of consensus nationally about appropriate visitation policy indicates there was equipoise. Randomized trials have been run in the past and more can be run to test the effectiveness of various policies for reducing numbers and/or hours of visits, for example, to establish whether there is a benefit of these policies on inpatient viral transmission. These trials should ideally include secondary endpoints looking at overall patient health, including all-cause mortality, length of hospital stay and countervailing effects, such as detrimental impacts on family members, healthcare workers and effects on healthcare spending.

One review of observational studies (Hugelius et al. 2021) has outlined numerous negative consequences of COVID-19 visitor restrictions. Associated effects on patients included increased pain, decreased nutritional intake, increased depressive symptoms and loneliness. For family members, restrictions were associated with increased anxiety, family relations disturbances and decreased bonding with newborns. Healthcare workers faced the stress of additional ethical dilemmas, expectations to implement remote visit options and worsening health of patients lacking family and emotional support.

Even if risk of disease transmission is decreased, visitor restrictions may paradoxically increase the infection fatality rate (IFR) through their detrimental health impacts such as worsened nutritional status, depression and lack of bedside family to notice adverse reactions or rapid health status changes. If the IFR was in fact increased due to the restrictions, this would render these policies calamitous, especially considering nearly everyone has now been infected with SARS-CoV-2 at least once (Klassen et al. 2021).

While randomized studies can provide information regarding the risks of in-hospital viral transmission reduction, the new information should be viewed in light of ethical considerations. Even if high quality studies identify some benefit against viral spread, would this result be worth leaving patients entirely alone at critical moments of birth, life and death?

(2) Harming one patient because it may benefit others is not consistent with basic medical ethics

In general, it is viewed as medically unethical to harm a patient in your care because there is a chance another patient will benefit (Kreps and Monin 2014). Doing so is particularly difficult to defend if the chance a third party will benefit is not well established. This also raises the question of at which point restricting visitors becomes directly harmful to a patient and at what point it is a physician’s duty to speak out against the restriction policy, irrespective of potential consequences for other hospitalized patients.

Physicians have also raised concerns that families separated from patients due to visitor restrictions were unable to regularly assess their loved one’s clinical status, which impaired patient care (Wentlandt et al. 2023). Family members at bedside are often well-equipped to understand the patient’s current condition and preferences (Wentlandt et al. 2023).

(3) Policies enacted by healthcare administrators may be too narrowly focused

During COVID-19, administrators were required to make decisions under pressured and uncertain circumstances from their vantage point. It is important to consider whether hospital and long-term care facility administrators are best suited to set patient visitation policies. Administrators may prefer to have basic guidelines and a plan for shared decision making—as well as better data—when faced with the prospect of changing policies in the future.

Administrators are expected to err on the side of more restrictive policies to achieve specific medical outcomes or to avoid lawsuits for negative health outcomes. Patients, families and healthcare workers alike should be aware that policies set by hospitals and nursing homes are not inexorable and can and should be challenged if unnecessary harm is being done or it is felt human rights are being infringed upon. Challenges can either lead to more collaborative decision making or, when necessary, trigger legislative or administrative action, which may lead to more humane policies in the future.

(4) Fundamental patient rights should be legally protected

A number of countries and states in the United States have taken steps to legally protect the rights of patients to be with loved ones in the hospital and/or at the end of life. European nations (1994) and the United Kingdom (Department of Health and Social Care. COVID-19: Our action plan for adult social care 2020), for example, have established “the right to enjoy the support of family” and the “right to say good-bye”, respectively. Florida and Alabama recently established laws which guarantee in-person visitation by at least one person for at least 2 h per day in all healthcare facilities for end-of-life situations and all children (https://flgov.com/2022/04/06/governor-ron-desantis-signs-bill-to-guarantee-visitation-rights-for-patients-and-their-families/, 2023. Though these rights have not been formally legislated in other states, there are some laws which mention it is a hospital’s obligation to address a dying patient’s “psychosocial, emotional and spiritual needs”, which families and caretakers may cite if interested in defending their visitation rights (Sudai 2021).

(5) The loss of the ability to consent is a form of coercion

Dying patients and infants are not physically capable of leaving against medical advice and, during COVID-19, the most restrictive visitor policies meant all family members were physically barred from visiting these patients against their will. Coercion involves forceful actions which violate the free will of an individual in order to achieve a desired response or behavior. B.F. Skinner (1971) among others (Skinner 1971) considered coercion to include forcing someone to act against their will. Situations with a power imbalance that forces those with less power to comply with specific requirements against free will are considered forms of coercion in multiple philosophical frameworks (Skinner 1971).

There is a clear power imbalance in modern healthcare where patients and families must comply with facility policies in order to have access to critical and potentially life-saving care. While few would debate healthcare facilities should be allowed to have rules about a maximum number of visitors or have the right to keep visitors out who are an active threat to other patients, highly restrictive policies, including barring all visitors of dying patients, which may be manifestly detrimental and almost uniformly undesirable to patients and family members, can be viewed as highly coercive, because of the degree to which they conflict with patient and family wishes. It should be recognized that patients receiving life-sustaining or end-of-life care, along with their family members have little to no agency to oppose even very undesirable, unethical or objectively harmful policies which accompany their care. Healthcare facilities may consider involving multiple parties including bioethicists and patient advocates in an ongoing manner to avoid coercive or unethical policies. Hospitals may also consider allowing patients and families/loved ones to request urgent appeals to bioethicists and/or administrations to policies which are particularly problematic in individual circumstances. Finally, as discussed above, families and healthcare facilities alike may benefit from some patient visitor rights being protected by law.

(6) Moral injury and dehumanization in healthcare may result from unscientific or visibly harmful policies

Sixth, moral injury (Goltz 2020) to healthcare workers (Bill and to Guarantee Visitation Rights for Patients and their Families. 2022) can occur when workers are complicit in, bear witness to, or fail to prevent an event that violates their fundamental ethical beliefs. During the COVID-19 pandemic, some physicians expressed feeling powerless when their attempts to challenge restrictive policies or advocate for visitation rights failed (Wentlandt et al. 2023). Moreover, some physicians felt a heavy emotional burden when having to act as a “gatekeeper” responsible for upholding policies that limited visitation between patients and loved ones (Wentlandt et al. 2023). A palliative care physician described (Wentlandt et al. 2023), “It’s like there is this inhumanity to the whole interaction where you are speaking to someone who you’ve never met over the phone. And telling them and you’re acting as the gatekeeper, you no, no you can’t come in. No. No, yes. Only one son can come in but the other no.”

Additionally, medical students, nurses, physicians in training and other healthcare workers may have felt they were not in a position to prevent a patient or family from suffering from an unjust policy. This may have created guilt and moral injury. Moral injury can result in breakdown of family relationships and social networks as well as a person’s ability to function and relate to others at work (Dean et al. 2019).

Conversely, physicians, including those in training, who work under these conditions may become desensitized to policies they are exposed to on a daily basis or see implemented by their mentors. A superficial perception that the policies were acceptable and necessary may make young physicians more inclined to accept or promote similarly restrictive policies in the future without serious reflection about the broader effects.

Policies which devalue patients’ relationships with their families can also have a dehumanizing effect. Those experiencing or witnessing the most devastating hospital visitor bans may subconsciously come to view family and interpersonal relationships as less important than the mitigation of a specific disease. This may result in a shift in a society’s values, whereby family members and social contacts respect and value their relationships with others less.

In addition, and/or alternatively, those who suffer from these policies may understandably come to the conclusion that hospitals do not have their best interests in mind, even if their individual caretakers do. A shift in the locus of control of hospital policies from healthcare workers involved directly with patient care to more distanced administrators may result in care which increasingly carries net harm and/or alienates even larger segments of society from the healthcare system in general.

## Conclusion

Though some forms of visitor limitations may be appropriate for certain diseases, effectiveness should be demonstrated through randomized studies which also consider secondary impacts on health. These trials will help patients, their providers and individual hospitals make evidence-based decisions. However, any evidence of benefit should be weighed with the accompanying loss of patient and family autonomy. Protection of fundamental rights of dying patients, children and infants to be with family and parents could be established either at the individual healthcare facility level or through state or national legislation.

Current data do not support a net benefit of COVID-19 hospital visitor restrictions, with high-quality evidence being extremely limited. A failure to promptly develop a framework for evidence development and the protection of basic patient and family rights will likely lead to reinstatement of similar if not stricter restrictions for infectious diseases in the future.
